# Microcosting the optimization of PrEP implementation among sexual and gender minority individuals with a substance use disorder

**DOI:** 10.1186/s13722-026-00672-4

**Published:** 2026-05-04

**Authors:** Danielle Ryan, Cathy J. Reback, Raymond P. Mata, Michael Li, Ali Jalali, Philip J. Jeng, David Benkeser, Sean M. Murphy

**Affiliations:** 1 Department of Population Health Sciences, Weill Cornell, New York, NY USA; 2 Friends Research Institute, Inc, Los Angeles, CA USA; 3 Center for HIV Identification, Prevention and Treatment Services, Department of Family Medicine, University of California, Los Angeles, CA USA; 4 Center for Behavioral and Addiction Medicine, Department of Family Medicine, University of California, Los Angeles, CA USA; 5 Center for Health Economics of Treatment Interventions for Substance Use Disorder, HCV, and HIV (CHERISH), New York, NY USA; 6 Department of Biostatistics and Bioinformatics, Rollins School of Public Health, Emory University, Atlanta, GA USA

**Keywords:** Cost, PrEP, Sexual and gender minority individuals, Substance use disorder

## Abstract

**Aim:**

Substance Use Disorder (SUD) increases HIV risk and is often a barrier to advancement along the PrEP Care Continuum. In the United States ~ 40,000 new HIV diagnoses occur annually, the majority among sexual and gender minorities who use substances. Insufficiently treated HIV and SUD results in substantial costs to society. Effective strategies that focus on SUD as part of a client-centered approach to improve PrEP engagement and address social determinants of health (SDoH), are critical. Understanding resources/cost requirements is important to know for prospective sites.

**Methods:**

The parent R01 was a single-site, randomized stepped-care and cost-effectiveness trial of Assisted Service Knowledge (A.S.K)-PrEP vs. standard of care (SOC), where “non-responders” in the A.S.K-PrEP arm were stepped to additional attention for SUD via contingency management (CM), resulting in three strategies against SOC (A.S.K-PrEP; A.S.K-PrEP + CM; A.S.K-PrEPàCM alone). Resources needed to implement and sustain each strategy were captured as part of a detailed microcosting analysis. Resources were categorized as fixed start-up, time-dependent, or variable, and organized within the implementation or sustainment phase. Costs were assigned using nationally representative prices.

**Results:**

Start-up costs (first year costs) were $25,958 and time-dependent costs (annually based) were $68,442 across strategies. Variable costs (annually based) differed across strategies resulting in $31,716 for A.S.K-PrEP alone, $31,994 for A.S.K-PrEP + CM and $30,271 for CM alone. The per-participant intervention cost was $55 for Standard of Care, $104 for A.S.K-PrEP alone, $73 for A.S.K-PrEP + CM, and $16 for CM alone.

**Conclusion:**

This study provides key insights into the “real-world” resource/cost requirements for implementing and sustaining each A.S.K.-PrEP strategy. Our results and publicly available budget impact tool provide support for potential stakeholders to ensure both feasibility and long-term sustainability for PrEP uptake and adherence for sexual and gender minority individuals with a SUD.

**Trial registration number:**

NCT05934877 and was registered on 05/23/23 as a clinical trial.

## Background

The current prevalence of HIV in the United States among individuals aged 13 and older is below 0.5%; [1] however, gender and sexual minority populations (i.e., transgender women and sexual minority men) face far greater risk of infection. Gender minority women, specifically women who identify along the trans feminine spectrum, currently experience the greatest burden for HIV, with an estimated prevalence range of 22%-28% [2], while sexual minority cisgender men (SMM) have an estimated prevalence of 15% [3]. Highly effective pre-exposure prophylaxis (PrEP) medications to prevent HIV are available, including long-acting injectables [4]. Among the largest barriers to PrEP are initiation, adherence, and persistence in individuals with a substance use disorder (SUD) [5–7]. 

The average lifetime healthcare cost per person living with HIV is ~$368,000, but rises to $441,600 -$588,800 for those with comorbid SUD [8]. SUD is associated with reduced quality-of-life, as well as increased risk of overdose mortality, excess utilization of high-cost healthcare services, engagement in a street economy, and incarceration [9–16]. Therefore, the average lifetime cost of non-healthcare SUD related consequences could be another ~$2 million per person [17]. Thus, efforts to strengthen PrEP engagement alongside SUD care are fundamental to individual health, public well-being, and national economic stability.

Assistance Services Knowledge-PrEP (A.S.K-PrEP) is a client-centered PrEP navigation intervention supported by text messages tailored to gender and sexual minorities with a SUD, as well as a stepped-care component where “non-responders” receive additional attention for their SUD via incorporation of contingency management (CM), given its strong foundation in the treatment of SUDs, particularly stimulant use disorder [18, 19]. CM refers to a type of behavioral therapy in which individuals are ‘reinforced’, or rewarded, for evidence of positive behavioral change [20]. The objective of this study was to identify the resources and estimate the associated costs required to implement and sustain the A.S.K-PrEP intervention in a “real-world” setting. Well-informed resource and cost requirements enhance the implementation of new interventions by supporting accurate budgeting, and efficient resource allocation.

## Methods

### A.S.K.-PrEP overview

**Fig. 1:**
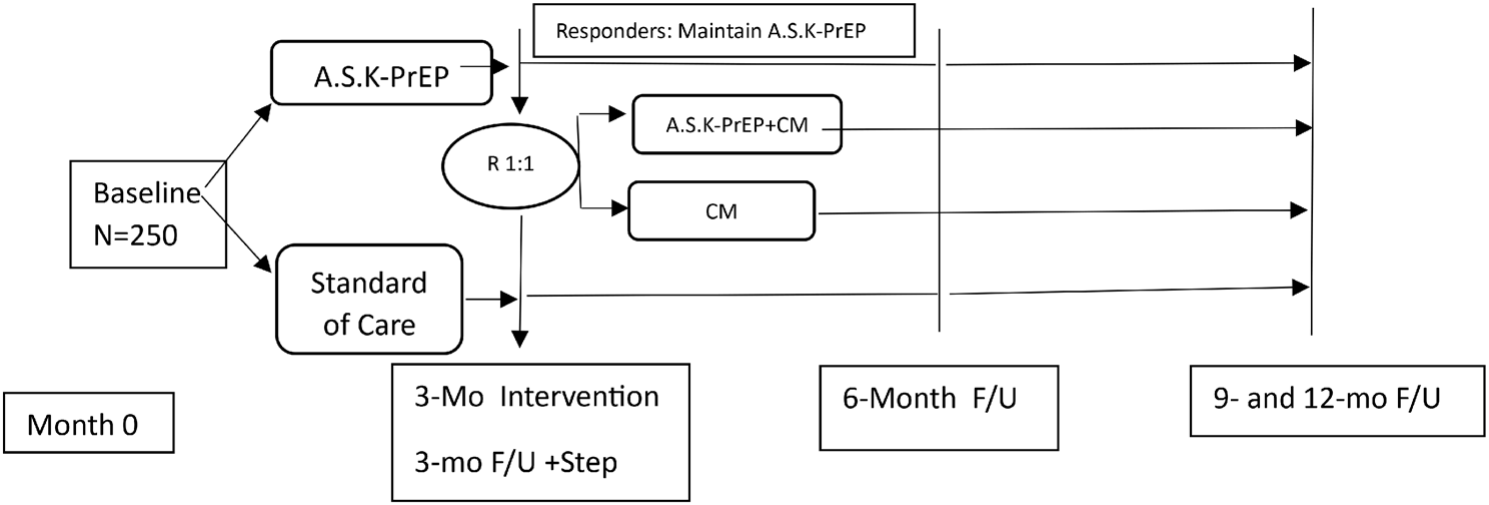
Schematic of study design. Reback CJ, Landovitz RJ, Benkeser D, Jalali A, Shoptaw S, Li MJ, et al. Protocol for a randomized controlled trial with a stepped care approach, utilizing PrEP navigation with and without contingency management, for transgender women and sexual minority men with a substance use disorder: Assistance Services Knowledge-PrEP (A.S.K.-PrEP). Addict Sci Clin Pract. 2024;19(1):79

The parent R01, AS.K.-PrEP, was a two-arm randomized controlled trial, with a stepped care approach, among HIV-negative sexual and gender minority individuals with an SUD who reside in Los Angeles County (LAC) aged 18 or older. Participants were randomized (3:1) to the A.S.K-PrEP Stepped Care arm or Standard of Care (SOC), respectively. Those randomized to the SOC arm received an educational session on PrEP, a PrEP pamphlet, and a list of clinics that provide PrEP in LAC. Participants in the A.S.K-PrEP stepped-care arm received 3 months of client-centered PrEP navigation (5 sessions), supplemented with weekly text-messaging support. At 3-months, participants were assessed on PrEP initiation, adherence, and use of their targeted substance (identified at baseline). “Non-responders” were stepped and re-randomized (1:1) to CM alone, or CM with an additional 3 months of PrEP navigation and text support. Over the course of 12 weeks, participants met with a behavioral technician thrice weekly for urinalysis and received voucher points for urine samples nonreactive to their targeted substance (see Fig. 1). Participants in a CM arm were rewarded $2.50 voucher points for the first non-reactive urine sample, which increases by $0.50 voucher points for each subsequent non-reactive urine sample. If a participant provided three consecutive nonreactive urine samples, a bonus voucher of $7.50 could be given weekly for 12 weeks. Voucher points, valued at $1.00, were redeemable at any time during the study for items or services that encourage a pro-social and healthy lifestyle (e.g., clothes, groceries, bicycle, paying bills but not cigarettes, alcohol, and firearms). A positive urine sample placed the participant at the initial voucher point value; however, the participant was able to return to their place in the schedule following three consecutive nonreactive urine samples [21]. All study procedures were approved by the Western Institutional Review Board (IRB Study # 1338919; IRB Tracking # 20224309). The study was registered as a clinical trial (NCT05934877). Additional and detailed study procedures have been reported elsewhere [22].

### Data collection

A detailed microcosting analysis was conducted to capture the resources that would be required to implement and deliver each intervention in a “real-world” setting [23]. Non-intervention costs, such as PrEP medications, were excluded from the analysis, as they were not part of the A.S.K-PrEP intervention. The focus of the microcosting analysis was the “real-world” resource/cost requirements to deliver A.S.K-PrEP. The intervention was delivered at a local community-based organization, where participants were connected to nearby providers for PrEP initiation and monitoring. The activity-based costing (ABC) method was used to accurately collect data about each activity [24–26]. The process included a site-visit followed by semi-structured interviews with personnel involved in the day-to-day operations relevant to each intervention, which included PrEP navigators, a clinical director, the clinical manager, and behavioral technicians (cross-trained as phlebotomists). A follow-up interview was conducted virtually to confirm initial findings and determine whether any additional process changes has occurred. One of the objectives was to estimate the clients that can be served at a given point in time with the resources available. The number of clients served under trial restrictions would have artificially inflated the results, therefore an average amount of clients were determined based on operational capacity to reflect a real world setting. Nationally representative price weights were assigned to each resource and are reported in Table 1. The average, per-participant, total payout for CM was estimated according to the amounts received by participants who had been stepped-up to receive either CM or A.S.K-PrEP + CM and had completed the 12-week CM reinforcement schedule at the time the microcosting analysis was conducted (*n* = 5).

**Table 1:** Nationally representative unit costs for A.S.K-PrEP supplies and labor

Supplies	Unit Cost	Quantity	$ 5,853
Phone	$133	1	$ 133
Cell Phones	$50	3	$ 150
Tablets	$1,468	2	$ 2,936
Computer	$717	2	$ 1,434
Online Marketing Advertisements	$1,000	1	$ 1,000
Online Impressions	$100	2	$ 200
**Employee**	**Salary**	**Fringe**	**Adjusted Annual Salary**
Clinical Supervisor	$119,460	31%	$156,493
Prevention Services Manager	$119,460	31%	$156,493
Behavioral Technician	$60,130	31%	$78,770
PrEP Navigator	$54,220	31%	$71,028

Source: https://www.gsaadvantage.gov/advantage/ws/catalog

Source: https://www.bls.gov/oes/current/oes_stru.htm. Personnel salaries sourced from the U.S. Bureau of Labor Statistics Occupational Employment and Wage Statistics (https://www.bls.gov/oes). A 31% fringe benefit rate was applied to all personnel costs

### Data analysis

Resources/costs were organized within a modified version of our existing budget impact tool [27], and reviewed with the site for accuracy. To ensure relevance, resources were organized to align with typical budget cycles of 3–5 years [28]. Resources were identified as *fixed start-up*, *time-dependent*, or *variable*, and categorized into one of two phases: *implementation* or *sustainment*. *Fixed start-up* resources are one-time commitments needed to establish an intervention; *time-dependent* resources are recurring but fixed over a given amount of time; and *variable* resources are those directly tied to the care of each client. The implementation phase was considered to be the period from the inception of the intervention, including planning activities, until steady-state; thus, it consists of resources utilized in all three categories over the timeframe of interest. It is assumed that the site would distribute the fixed-cost payments over a 12-month duration of the implementation period, leaving only *time-dependent* and *variable* resources for the duration of the sustainment period.

## Results

The implementation phase for A.S.K-PrEP was determined to be 12 months. *Fixed start-up* resources included supplies, outreach, and one-time trainings for personnel. *Time-dependent* resources/costs included monthly fees associated with text messages, laboratory costs, and phones; weekly and bi-weekly meetings; and annual trainings. The text messages and laboratory costs do not differ by the number of individuals and were purchased on a monthly basis. The variable resources required to implement the intervention in addition to SoC included time needed to complete necessary forms (intake, locator form), PrEP navigator sessions, CM, and non-participant-facing activities, such as scheduling, screening, and programming texts (see Table 2. Microcosting Dashboard).

**Table 2:** A.S.K-PrEP microcosting dashboard: program-level implementation and sustainment costs (2024 USD)

Resource Category		
Start Up Costs		$25,958
	Equipment/Supplies	$5,853
	Online Advertising	$13,075
	Training	$7,030
Time Dependent Costs		$68,442
	Rent	$59,280
	Monthly Fees:	
	Texting Application	$120
	Phone	$1,800
	Intervention Laboratory Costs	$684
	Meetings	$6,320
	Trainings	$238
Variable Costs		$30,924
	Intake	$4,380
	Standard of Care	$409
	Needs Based Assessments	$453
	Contingency Management	$69
	Patient Contact	$24,307
	Supplies	$1,305
Total Costs during year 1		$125,325
Total Costs during subsequent years		$99,366
Annual Per-Participant Costs		
	Randomized to Standard of Care	$55
	Randomized to A.S.K-PrEP	$104
	Re-randomized to A.S.K-PrEP+Contingency Management	$73
	Re-randomized to Contingency Management alone	$16

Notes: Per-participant costs reflect the incremental cost per participant per completed strategy. Non-intervention costs (e.g., PrEP medications) are excluded; intervention laboratory costs include phlebotomy kits for HIV status and PrEP adherence monitoring and urine drug screens only. A.S.K.-PrEP: Assistance Services Knowledge-PrEP; CM: Contingency Management; SOC: Standard of Care; SUD: Substance Use Disorder

The fixed start-up cost was estimated to be $25,958, and was largely driven by outreach, which consisted of 2 behavioral technicians who contributed approximately 16 h per month for 12 months. Outreach activities included passing out flyers and contacting other organizations. A one-time purchase of equipment included phones (desk and cellular), tablets, computer, and online marketing. The behavioral technicians had one-time trainings for HIV testing, counseling, and bloodborne pathogens. PrEP navigators had 2 weeks of providing non-clinical support services (i.e., help to develop recovery plans, effective coping habits, and life skills), training (~ 80 h), and an 8-hour motivational interviewing training that was led by a clinical manager. The PrEP navigators and behavioral technicians both received a roughly 8-hour introductory training on PrEP that entailed information on the available medications, appropriate populations for each medication type, where PrEP can be accessed in LAC, and the benefits of initiating and adhering to PrEP [29]. 

The time-dependent costs were $68,442. These costs included rent for a 130 square foot space in LAC, situated in a transitional neighborhood adjacent in Hollywood. The location is central to LAC’s culturally diverse sexual and gender minority communities and falls within the LAC’s Health Service District with the greatest incidence of HIV. Other time-dependent expenditures included monthly fees for an automated text messaging application, laboratory supplies, and phones. Supplies included phlebotomy kits for bloodwork (i.e., HIV status and PrEP adherence), urine screens to determine current drug use, as these services were provided at the intervention site; and phones to contact participants. PrEP navigators also met twice a month with a clinical supervisor for an hour, and once a week with the prevention service manager for about 30 min. The purpose of these meetings was to discuss participants who might require additional support or had been absent from scheduled sessions. The behavioral technicians were required to complete bi-annual phlebotomy trainings, and an annual HIV certification.

The variable costs were approximately $31,333. All participants met with a behavioral technician to fill out intake and locator forms, and complete laboratory work (blood and urine). Under SOC a participant would spend approximately 15 min with the behavioral technician to learn about PrEP and how to access PrEP services in the area. Participants in the A.S.K-PrEP intervention arm spent approximately one hour meeting with a PrEP navigator initially, regarding access to PrEP and social determinant of health (SDoH) barriers to PrEP initiation and adherence (e.g., shelter, clothing, food, transportation). Subsequent sessions lasted approximately 30 min and were used primarily to address new barriers that had arisen. At the 3-month assessment, participants who did not initiate or adhere to PrEP would then be stepped-up and re-randomized 1:1 to either A.S.K-PrEP + CM, or CM alone. The CM introduction session was approximately 10 min and the subsequent CM visits were approximately 15 min. The average CM payout per participant was $75 for the 3-month CM intervention. PrEP navigators and behavioral technicians spent approximately 10 to 16 additional hours per month contacting participants for intake, screening, and follow-ups.

The operational capacity and monthly client engagement during the steady-state period was an average of 8 clients could be served per month, given the resources available at the time, and the absence of study-specific requirements. The estimated total cost for the first year of the intervention (i.e., the implementation phase) was $125,325 followed by annual sustainment costs of $99,366, amounting to an average annual per-client costs of $1,035, respectively.

## Discussion

The successful adoption of new interventions requires careful resource/budget planning across all stages (i.e., design through maintenance). Uncertainty among decision-makers regarding the short-term financial and logistical demands of an intervention can introduce unnecessary barriers to implementation of effective interventions, which can be alleviated via microcosting estimates. The annual per-patient costs do not vary greatly due to major costing components being similar across intervention strategies. The additional resources required for the A.S.K PrEP strategies are relatively low to SoC. The intervention strategies range from $16-$104. Initially, clients are randomized to only two intervention strategies and information such as an intake assessment and a locator form are taken. If clients are not responsive then they are re-randomized to A.S.K PrEP + CM or CM only, which decreases the amount of time and resources, given that details on the client have been established earlier in the intervention.

To our knowledge this is the first microcosting analysis for a client-centered PrEP navigation intervention designed specifically for gender and sexual minorities with a SUD. Recent literature on estimating HIV prevention costs is limited and varies on PrEP related services, such as delivery methods in clinical based settings. For example, a systematic review assessed the costs of non-surgical HIV prevention interventions where only a handful collected microcosting data [30]. The systematic review found that the most used study design to be model-based economic evaluations. Only nine studies collected primary data where seven studies took a bottom-up approach and two studies used a top-down gross costing method, however “real-world” program delivery mechanisms and costs of intervention delivery were rarely considered [30]. These studies evaluated costs that were clinically based of different genders and countries, For example, Eakle et al. evaluated service delivery for PrEP in South Africa and found the cost for per person year for PrEP was $168 (inflated from 2015 dollars) [31]. Our publicly available budget impact tool can be utilized for different types of interventions to inform decision makers on sustainable costs and is published and accessible from the CHERISH repository www.CHERISHresearch.org.

### Strengths and Limitations

The primary strength of our analysis centers around the prospective design of the economic evaluation alongside the A.S.K-PrEP intervention, including the microcosting analysis, while using nationally representative costs to address generalizability. This approach enabled a comprehensive assessment of all intervention components, leveraging insights from site leadership and staff to contextualize resource needs, operational constraints, and implementation challenges in a “real-world” setting. However, a notable limitation is that the study was conducted in an urban setting on the West Coast of the United States and may not fully represent of the HIV and substance use co-epidemic, and the impacted communities in other regions of the US.

## Conclusion

PrEP is a highly effective prophylaxis against HIV infection; yet, the persistently high rates of HIV infection among sexual and gender minority individuals underscore the importance of interventions that not only facilitate PrEP uptake, but also provide targeted support to overcome structural and behavioral barriers to adherence and retention such as SUD. A.S.K-PrEP represents a promising model that leverages local community-based resources for integrating PrEP navigation with support for SUD, to improve health outcomes among those at highest risk.

Our findings provide key insights into the resource and cost requirements necessary to implement and sustain A.S.K-PrEP in a “real-world” setting. Additionally, the accompanying publicly available budget impact tool enables potential stakeholders to consider the financial implications of incorporating A.S.K-PrEP into their unique settings.

## Data Availability

Upon Request.

## References

[1] HIV Surveillance Supplemental Report. Estimated HIV Incidence and Prevalence in the United States, 2018–2022 [05/21/2024]. Available from: https://stacks.cdc.gov/view/cdc/156513.

[2] Baral SD, Poteat T, Stromdahl S, Wirtz AL, Guadamuz TE, Beyrer C. Worldwide burden of HIV in transgender women: a systematic review and meta-analysis. Lancet Infect Dis. 2013;13(3):214–22. 23260128 10.1016/S1473-3099(12)70315-8

[3] Beyrer C, Baral SD, van Griensven F, Goodreau SM, Chariyalertsak S, Wirtz AL, et al. Global epidemiology of HIV infection in men who have sex with men. Lancet. 2012;380(9839):367–77. 22819660 10.1016/S0140-6736(12)60821-6 PMC3805037

[4] HIV Prevention: Pre-Exposure Prophylaxis (PrEP) [Available from: https://hivinfo.nih.gov/understanding-hiv/fact-sheets/pre-exposure-prophylaxis-prep].

[5] Grant RM, Lama JR, Anderson PL, McMahan V, Liu AY, Vargas L, et al. Preexposure chemoprophylaxis for HIV prevention in men who have sex with men. N Engl J Med. 2010;363(27):2587–99. 21091279 10.1056/NEJMoa1011205 PMC3079639

[6] Goodman-Meza D, Beymer MR, Kofron RM, Amico KR, Psaros C, Bushman LR, et al. Effective use of pre-exposure prophylaxis (PrEP) Among stimulant users with multiple condomless sex partners: a longitudinal study of men who have sex with men in Los Angeles. AIDS Care. 2019;31(10):1228–33. 30894013 10.1080/09540121.2019.1595523 PMC6663637

[7] Hoenigl M, Jain S, Moore D, Collins D, Sun X, Anderson PL, et al. Substance Use and Adherence to HIV Preexposure Prophylaxis for Men Who Have Sex with Men(1). Emerg Infect Dis. 2018;24(12):2292–302. 30457536 10.3201/eid2412.180400 PMC6256399

[8] Bingham A, Shrestha RK, Khurana N, Jacobson EU, Farnham PG. Estimated Lifetime HIV-Related Medical Costs in the United States. Sex Transm Dis. 2021;48(4):299–304. 33492100 10.1097/OLQ.0000000000001366

[9] Anderson-Carpenter KD, Fletcher JB, Reback CJ. Associations between Methamphetamine Use, Housing Status, and Incarceration Rates among Men Who Have Sex with Men and Transgender Women. J Drug Issues. 2017;47(3):383–95. 28670005 10.1177/0022042617696917 PMC5485860

[10] Bockting WO, Miner MH, Swinburne Romine RE, Hamilton A, Coleman E. Stigma, mental health, and resilience in an online sample of the US transgender population. Am J Public Health. 2013;103(5):943–51. 23488522 10.2105/AJPH.2013.301241 PMC3698807

[11] Bowers JR, Branson CM, Fletcher J, Reback CJ. Differences in substance use and sexual partnering between men who have sex with men, men who have sex with men and women and transgender women. Cult Health Sex. 2011;13(6):629–42. 21442499 10.1080/13691058.2011.564301

[12] Bowers JR, Branson CM, Fletcher JB, Reback CJ. Predictors of HIV Sexual Risk Behavior among Men Who Have Sex with Men, Men Who Have Sex with Men and Women, and Transgender Women. Int J Sex Health. 2012;24(4):290–302. 24660042 10.1080/19317611.2012.715120 PMC3960284

[13] Brewer RA, Magnus M, Kuo I, Wang L, Liu TY, Mayer KH. The high prevalence of incarceration history among Black men who have sex with men in the United States: associations and implications. Am J Public Health. 2014;104(3):448–54. 24432948 10.2105/AJPH.2013.301786 PMC3953792

[14] Kussin-Shoptaw AL, Fletcher JB, Reback CJ. Physical and/or Sexual Abuse Is Associated with Increased Psychological and Emotional Distress Among Transgender Women. LGBT Health. 2017;4(4):268–74. 28498023 10.1089/lgbt.2016.0186 PMC5564039

[15] Reisner SL, Bailey Z, Sevelius J. Racial/ethnic disparities in history of incarceration, experiences of victimization, and associated health indicators among transgender women in the U.S. Women Health. 2014;54(8):750–67. 25190135 10.1080/03630242.2014.932891 PMC5441521

[16] Valentine SE, Peitzmeier SM, King DS, O’Cleirigh C, Marquez SM, Presley C, et al. Disparities in Exposure to Intimate Partner Violence Among Transgender/Gender Nonconforming and Sexual Minority Primary Care Patients. LGBT Health. 2017;4(4):260–7. 28719246 10.1089/lgbt.2016.0113

[17] Murphy SM. The cost of opioid use disorder and the value of aversion. Drug Alcohol Depend. 2020;217:108382. 33183909 10.1016/j.drugalcdep.2020.108382 PMC7737485

[18] Hughto JMW, Quinn EK, Dunbar MS, Rose AJ, Shireman TI, Jasuja GK. Prevalence and Co-occurrence of Alcohol, Nicotine, and Other Substance Use Disorder Diagnoses Among US Transgender and Cisgender Adults. JAMA Netw Open. 2021;4(2):e2036512. 33538824 10.1001/jamanetworkopen.2020.36512 PMC7862992

[19] De Crescenzo F, Ciabattini M, D’Alo GL, De Giorgi R, Del Giovane C, Cassar C, et al. Comparative efficacy and acceptability of psychosocial interventions for individuals with cocaine and amphetamine addiction: A systematic review and network meta-analysis. PLoS Med. 2018;15(12):e1002715. 30586362 10.1371/journal.pmed.1002715 PMC6306153

[20] Petry NM. Contingency management: what it is and why psychiatrists should want to use it. Psychiatrist. 2011;35(5):161–3. 22558006 10.1192/pb.bp.110.031831 PMC3083448

[21] Higgins ST, Budney AJ, Bickel WK, Hughes JR, Foerg F, Badger G. Achieving cocaine abstinence with a behavioral approach. Am J Psychiatry. 1993;150(5):763–9. 8480823 10.1176/ajp.150.5.763

[22] Reback CJ, Landovitz RJ, Benkeser D, Jalali A, Shoptaw S, Li MJ, et al. Protocol for a randomized controlled trial with a stepped care approach, utilizing PrEP navigation with and without contingency management, for transgender women and sexual minority men with a substance use disorder: Assistance Services Knowledge-PrEP (A.S.K.-PrEP). Addict Sci Clin Pract. 2024;19(1):79. 39521970 10.1186/s13722-024-00482-6 PMC11549772

[23] Neumann P, Sanders G, Russell L, Siegel J, Ganiats T. Cost-Effectiveness in Health and Medicine 2. OUP; 2017.

[24] Chapko MK, Liu CF, Perkins M, Li YF, Fortney JC, Maciejewski ML. Equivalence of two healthcare costing methods: bottom-up and top-down. Health Econ. 2009;18(10):1188–201. 19097041 10.1002/hec.1422

[25] Negrini D, Kettle A, Sheppard L, Mills GH, Edbrooke DL. The cost of a hospital ward in Europe: is there a methodology available to accurately measure the costs? J Health Organ Manag. 2004;18(2–3):195–206. 15366283 10.1108/14777260410548437

[26] Spacirova Z, Epstein D, Garcia-Mochon L, Rovira J, Olry de Labry Lima A, Espin J. A general framework for classifying costing methods for economic evaluation of health care. Eur J Health Econ. 2020;21(4):529–42. 31960181 10.1007/s10198-019-01157-9 PMC8149350

[27] Ryan DA, Montoya ID, Koutoujian PJ, Siddiqi K, Hayes E, Jeng PJ, et al. Budget impact tool for the incorporation of medications for opioid use disorder into jail/prison facilities. J Subst Use Addict Treat. 2023;146:208943. 36880906 10.1016/j.josat.2022.208943 PMC10084043

[28] Sullivan SD, Mauskopf JA, Augustovski F, Jaime Caro J, Lee KM, Minchin M, et al. Budget impact analysis-principles of good practice: report of the ISPOR 2012 Budget Impact Analysis Good Practice II Task Force. Value Health. 2014;17(1):5–14. 24438712 10.1016/j.jval.2013.08.2291

[29] AIDS Education & Training Center. Pre-exposure Prophylaxis. [Available from: https://aidsetc.org/topic/pre-exposure-prophylaxis].

[30] Bozzani FM, Terris-Prestholt F, Quaife M, Gafos M, Indravudh PP, Giddings R, et al. Costs and Cost-Effectiveness of Biomedical, Non-Surgical HIV Prevention Interventions: A Systematic Literature Review. PharmacoEconomics. 2023;41(5):467–80. 36529838 10.1007/s40273-022-01223-w PMC10085926

[31] Eakle R, Gomez GB, Naicker N, Bothma R, Mbogua J, Cabrera Escobar MA, et al. HIV pre-exposure prophylaxis and early antiretroviral treatment among female sex workers in South Africa: Results from a prospective observational demonstration project. PLoS Med. 2017;14(11):e1002444. 29161256 10.1371/journal.pmed.1002444 PMC5697804

